# Clinician-Centered Evaluation Framework for Explainable AI Heatmaps in OCT-Based Retinal Disease Classification

**DOI:** 10.3390/jimaging12050211

**Published:** 2026-05-16

**Authors:** Eirini Maliagkani, Ilias Georgalas, Ioannis Datseris, Elpiniki Papageorgiou, Ioannis D. Apostolopoulos

**Affiliations:** 1 1st Department of Ophthalmology, General Hospital of Athens “G. Gennimatas”, National and Kapodistrian University of Athens, 11527 Athens, Greece; igeorgalas@yahoo.com; 2 OMMA Ophthalmological Institute of Athens, 11525 Athens, Greece; jodats13@me.com; 3 Artificial Intelligence, Computational Methods and Technological Applications (ACTA) Laboratory, Department of Energy Systems, University of Thessaly, Gaiopolis Campus, 41500 Larisa, Greece; elpinikipapageorgiou@uth.gr (E.P.); iapostolopoulos@uth.gr (I.D.A.)

**Keywords:** explainable artificial intelligence, optical coherence tomography, swin transformer, model interpretability, heatmaps, clinical evaluation, clinical plausibility, evaluation framework, ophthalmology, retinal disease classification

## Abstract

This study presents a two-phase framework for selecting clinically plausible explainable artificial intelligence (XAI) heatmaps for retinal optical coherence tomography (OCT) classification. A six-class Swin Transformer model was trained and validated using a combined dataset consisting of a subset of the public OCT-C8 dataset and private data from a Greek tertiary hospital and externally evaluated on an independent dataset from a private ophthalmological institute. Diagnostic performance was high, achieving 97% accuracy in cross-validation and 91.82% on external evaluation. In Phase 1, one ophthalmologist and one artificial intelligence (AI) specialist independently assessed 100 heatmaps per method based on visual quality and anatomical plausibility, reducing the candidate methods to three. In Phase 2, 21 specialists evaluated the selected methods across multiple cases using a five-point Likert scale reflecting agreement between highlighted regions and the model diagnosis. The proposed Token contRAST map (TRAST) achieved the highest ratings, followed by Gradient-weighted Class Activation Mapping (Grad-CAM++), while Cosine-Grad Fusion Map (CGFM) showed the lowest performance. These findings reflect clinical plausibility rather than direct model interpretability and indicate that effective XAI in OCT imaging requires not only technical performance but also structured expert evaluation. The proposed framework provides a practical approach for selecting explanation methods suitable for clinical use in ophthalmology.

## 1. Introduction

Accurate differentiation among retinal diseases on optical coherence tomography (OCT) is essential for appropriate clinical management, yet many conditions present with overlapping imaging features that make diagnosis challenging even for experienced clinicians. OCT is a non-invasive imaging modality that uses near-infrared light to generate high-resolution cross-sectional images of the retina [1]. It plays a central role in the diagnosis and monitoring of major retinal diseases, including age-related macular degeneration (AMD), central serous chorioretinopathy (CSR), retinal vein occlusion (RVO), diabetic retinopathy, and macular hole (MH). In recent years, artificial intelligence (AI) methods have shown strong potential for automated OCT classification, offering opportunities to support clinical decision-making and improve diagnostic efficiency [2,3].

Recent classification models for OCT imaging have moved beyond conventional convolutional backbones to transformer-based models that capture broader spatial context in images. This is particularly applicable to retinal diseases, where diagnostic evidence can be both focal and diffuse. A recent example is the use of Swin Transformer variants for multi-class OCT classification, showing that hierarchical transformer features can support discrimination across multiple retinal conditions [2,4].

However, diagnostic performance based on conventional metrics alone is not sufficient to ensure clinical trustworthiness. Clinicians must understand not only the predicted class, but also the image evidence that supports a given decision and the conditions under which a model may be reliable. This requirement has led to increasing interest in explainable artificial intelligence (XAI), alongside emerging guidance on how explanations should be evaluated and reported for clinical use [5,6].

Heatmap-based explanations are the most commonly used approach in medical imaging, as they align with how clinicians visually inspect images. These methods aim to highlight regions that contribute to the model’s prediction, potentially supporting human interpretation. However, a heatmap is not necessarily a faithful representation of the model’s reasoning. Different explanation methods can produce substantially different visualizations for the same prediction, depending on the underlying mechanism and model architecture [7]. Moreover, even when a heatmap appears visually plausible, it may fail important robustness criteria, such as sensitivity to model perturbations or reproducibility across architectures [8]. This creates a potential safety concern, as explanations may increase user confidence without improving decision quality.

These challenges are particularly pronounced in transformer-based models. Transformers provide multiple internal signals that can be used to generate heatmaps, including gradient-based activations, attention mechanisms, and token-level representation similarities. As a result, different explanation methods may highlight different regions for the same prediction, leading to divergent clinical interpretations. In the absence of a structured selection process, the choice of a heatmap is often driven by convenience or qualitative inspection, which is insufficient for multi-class OCT diagnosis.

This variability and lack of consistency across explainability methods highlight the need for a structured approach for selecting clinically plausible heatmaps. Despite the widespread use of heatmap-based explanations in OCT studies, there is currently no standardized framework for selecting which explanation methods are clinically plausible. In many studies, heatmaps are presented as illustrative examples without systematic evaluation, multi-expert validation, or comparison across alternative methods. At the same time, recent clinical AI guidelines emphasize the importance of assessing explanations in terms of both technical validity and clinical plausibility, including their alignment with domain knowledge and their interpretability for end-users [5].

The objective of this study was to develop and evaluate a structured, clinician-centered framework for selecting explainability heatmaps in transformer-based OCT classification. The proposed two-phase approach first applies a quality-control screening step to exclude visually weak or anatomically implausible heatmaps, followed by a multi-expert clinical evaluation assessing the consistency between highlighted regions and model predictions. The framework emphasizes clinical plausibility as a complementary dimension to quantitative evaluation, rather than a replacement for it. To our knowledge, this is among the first studies to systematically compare multiple explainability methods for transformer-based OCT classification using structured and independent multi-expert evaluation.

## 2. Materials and Methods

**Figure 1:**
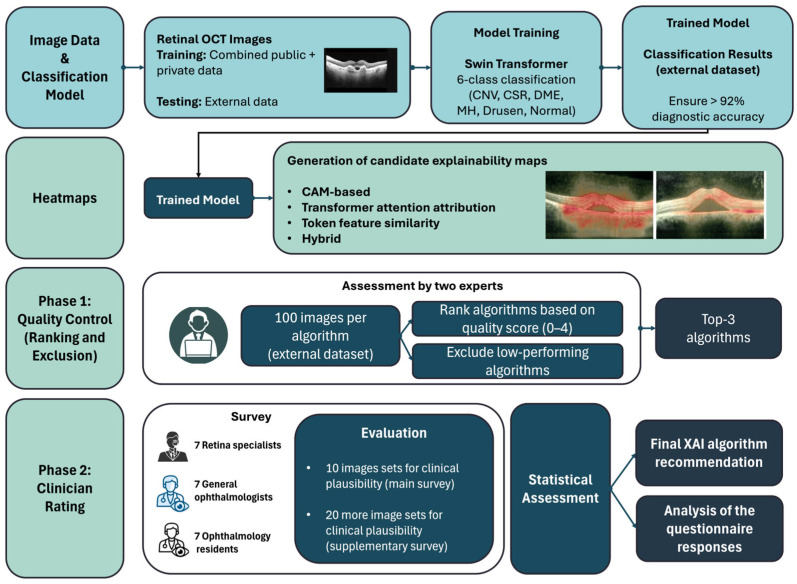
Overview of the methodology.

An overview of our methodology is provided in Figure 1. OCT images from a combined dataset (public OCT-C8 subset and private dataset) were used to train and cross-validate a six-class Swin Transformer model, which was subsequently evaluated on an independent private external dataset. Candidate explainability maps were then generated using class activation mapping (CAM)-based, attention-based, token-similarity, and hybrid methods. In Phase 1, candidate methods were screened for visual quality and for anatomically implausible highlighting. In Phase 2, the three selected methods were evaluated by ophthalmologists through a structured questionnaire-based assessment, followed by statistical analysis and final method comparison.

### 2.1. Datasets

The study included OCT B-scans from both public and private sources. The combined dataset used for model development consisted of a six-class subset of the publicly available OCT-C8 dataset (18,000 B-scans; 3000 per class), used with the original labels [9], and a private dataset (GEN), obtained from a Greek tertiary hospital (the General Hospital of Athens “G. Gennimatas”), comprising 334 routine clinical OCT B-scans. These two datasets were combined and used for model training and repeated 10-fold stratified cross-validation. The GEN dataset was included in the cross-validation folds together with the public dataset, ensuring that all data sources contributed to both training and validation across folds.

An independent external dataset (OMM), collected from a private eye institute in Greece (the OMMA Ophthalmological Institute of Athens), was used exclusively for model evaluation and explainability assessment. This dataset included 306 OCT B-scans reflecting real-world clinical variability and was not used during training, validation, or hyperparameter tuning.

The datasets included cases of choroidal neovascularization (CNV), CSR, diabetic macular edema (DME), drusen, MH, and normal retina. The public dataset was class-balanced (3000 images per class), whereas the private datasets reflected real-world clinical distributions (Table 1).

Both private datasets were independently annotated by four expert ophthalmologists (two retina specialists, one general ophthalmologist, and one ophthalmology resident).

All OCT B-scans were converted to grayscale float arrays in [0, 1], corrected for image orientation, resized by aspect-ratio-preserving LANCZOS letterboxing to 256 × 256 pixels, zero-padded where required, and replicated to three channels for compatibility with the ImageNet-pretrained SwinV2 backbone. Contrast harmonization used CLAHE with clip limit 2.0 and 8 × 8 tiles, followed by robust intensity normalization using the 2nd and 98th percentile range. Model inputs were normalized with ImageNet mean [0.485, 0.456, 0.406] and standard deviation [0.229, 0.224, 0.225].

Training augmentation consisted of random horizontal flip (*p* = 0.5), random rotation up to 15 degrees, random affine translation up to 3% in each axis with scale 0.89–1.15, brightness and contrast jitter of 0.3, random gamma adjustment with gamma sampled uniformly from 0.8 to 1.2 (*p* = 0.7), and Gaussian blur with kernel size 3 (*p* = 0.05). A detailed summary of the preprocessing and augmentation pipeline is provided in Supplementary Table S1.

### 2.2. Image Classification Model

The Swin Transformer is a hierarchical vision transformer (ViT) architecture [10] combining the global modeling capacity of self-attention [11] with the computational efficiency needed for high-resolution images. It works by partitioning the input image into non-overlapping local windows and then applying self-attention within each window [12]. It also introduces shifted window attention, where the window partitioning is offset between successive blocks [10]. The latter allows cross-window interactions while preserving the multi-stage feature hierarchy that progressively captures fine-to-coarse structures, which is particularly relevant to OCT imaging.

Swin was initialized with ImageNet-pretrained weights [13] and configured to output a single global feature vector per OCT image via average pooling. This feature vector was then passed to the classifier head, implemented as a shallow multilayer perceptron with one hidden layer, a non-linear activation, and dropout regularization, followed by a final linear layer that produced logits for the six retinal pathology classes. We kept the network’s head intentionally small to limit the number of randomly initialized parameters and ensure that most discriminative capacity remained in the pretrained transformer features. The backbone was initially frozen (untrainable), and training began by optimizing only the classifier head, after which progressively deeper transformer blocks were unfrozen. This particular procedure was adopted to preserve transferable low-level representations learned from prior general training (ImageNet), while allowing higher-level features to adapt to OCT-specific morphology. Late-stage normalization layers were also set to be trainable to support feature recalibration.

Optimization was performed with AdamW [14], which decouples weight decay from the gradient update and is commonly used for transformer training. We used a transfer-learning learning-rate scheme in which the pretrained backbone was updated with a smaller learning rate than the newly initialized classifier head. This allowed conservative updates in the backbone while enabling faster learning in the classifier head. The primary loss function was focal cross-entropy with label smoothing, which discourages overconfident predictions. Training incorporated an adaptive learning-rate scheduler that reduced the learning rate when validation accuracy plateaued, together with early stopping to prevent overfitting. The complete training configuration is summarized in Table 2.

The backbone parameter group used a learning rate of 3 × 10^−5^, whereas the classifier head used a learning rate of 3 × 10^−4^. Weight decay was 1 × 10^−4^. The loss function was focal cross-entropy with gamma = 2.0 and label smoothing = 0.05. Training used a maximum of 100 epochs with a minimum of 10 epochs before early stopping. Learning-rate scheduling used ReduceLROnPlateau to monitor validation accuracy. For cross-validation, the scheduler used factor = 0.5 and patience = 3, and early stopping patience was 10 epochs. For the external-validation/special protocol used by the final XAI checkpoint, the scheduler used factor = 0.8 and patience = 5, and early stopping patience was 12 epochs. Batch size was 16 in the standard cross-validation/full-training pipeline and 32 in the special external-validation protocol. Training was performed using Python 3.11.14, PyTorch 2.5.1, torchvision 0.20.1, timm 1.0.22, CUDA 12.1, and an NVIDIA GeForce RTX 3080 Ti GPU with 12 GB memory.

In this work, Swin serves as the selected classification carrier rather than the main methodological contribution. We used the official PyTorch ecosystem implementation available through the timm library [15], which provides standardized model definitions and training utilities for transformer backbones. The network was configured for six-class classification by adding a task-specific multilayer perceptron head.

### 2.3. Explainability Maps

Explainability heatmaps were derived by pooling multiple algorithms and performing the necessary adjustments for application to the Swin model. Table 3 summarizes the selected XAI algorithm families, methods, and variants used in the study. Detailed information on each algorithm is given in the following sub-sections and in the Supplementary Table S2. A short description of each algorithm is given in the following sub-sections.

For each XAI method, heatmaps were generated for the model-predicted class so that the explanation corresponded to the same decision evaluated by the clinician. All maps were min-max normalized within each image and upsampled to the 256 × 256 input resolution before visualization. For Grad-CAM, Grad-CAM++, and HiRes-CAM, the implementation selected the target layer using a backbone-aware search. Eigen-CAM and Score-CAM used the final SwinV2 LayerNorm representation. Score-CAM was restricted to the 64 channels with the largest activation variance to make computation tractable. Self-attention attribution used a hook on the final Swin attention softmax. EL-GWAA used gradient-weighted attention probabilities captured at the attn_drop input of the first two and last two Swin blocks, with early and late weights of 0.4 and 0.6, respectively. TRAST used token descriptors captured from the normalization outputs of the first two and last two Swin blocks, with norm2 preferred and norm1 used as a fallback when required. Target layers, hooks, normalization procedures, and method-specific implementation settings are summarized in Supplementary Table S2.

#### 2.3.1. CAM-Based

We used five CAM-based algorithms. The CAM family is implemented in a backbone-aware manner. When a clear convolutional feature map is available, the gradient-weighted class activation mapping (Grad-CAM) pipeline is applied to the last convolutional layer, which matches the original Grad-CAM assumptions. It also provides a compact spatial grid that can be interpolated back to the input image size [16].

Grad-CAM produces a class-conditional localization map by backpropagating the score of the target class to a selected late convolutional feature map. The gradients are spatially pooled to obtain one scalar weight per channel. The final heatmap is formed as the rectified, channel-weighted sum of the feature map. The implementation uses the predicted class as the default target to ensure that the explanation corresponds to the model’s decision, and upsamples the heatmap to the input resolution using bilinear interpolation [16].

Grad-CAM++ extends Grad-CAM by replacing the single global weight per channel with a more expressive weighting that uses higher-order gradient information. This improves localization in cases where multiple instances or distributed evidence contribute to the class score. The key difference from baseline Grad-CAM is how channel contributions are weighted and not how the final heatmap is post-processed or visualized [17].

Score-CAM is gradient-free and estimates channel importance through controlled perturbations. For each selected activation channel, its spatial activation pattern is upsampled to the input size, normalized to a unit mask, and used to multiplicatively gate the input image. To make Score-CAM tractable for high-dimensional transformer feature tensors, the implementation restricts computation to a subset of channels selected by the highest activation variance, which preserves the most spatially informative channels while substantially reducing runtime [18].

Eigen-CAM is also gradient-free and extracts a saliency map from the dominant variation in the feature representation. The selected feature tensor is flattened across spatial positions and decomposed with a singular-value decomposition. The first principal component is interpreted as the most informative direction of spatial variation. Eigen-CAM provides a class-agnostic structural explanation of what the network encodes strongly in the chosen layer and is class-agnostic [19].

HiRes-CAM is a CAM-family method that aims to preserve fine spatial detail by avoiding the gradient-averaging step used in baseline Grad-CAM. It forms the explanation map by combining feature-map activations with their spatially resolved backpropagated gradients and then aggregates across channels to obtain a class-specific relevance map [20].

#### 2.3.2. Transformer Attention Attribution

Transformer attention attribution (TAA) methods explain decisions by making use of the attention mechanism as a structured routing of information between tokens [21]. For the present Swin implementation, attention is computed within local windows and is cyclically shifted between blocks. The methods in this family compute attention-based relevance using gradient weighting, so that only attention connections that contribute positively to the target class score are emphasized. The resulting token importances are then converted to spatial heatmaps, aligned to the input resolution, and min–max normalized.

The self-attention baseline targets the attention probabilities of the final attention module in the backbone, since this block represents the highest-level token interactions immediately before classification [22].

Early late gradient-weighted window attention aggregation (EL-GWAA) extends the attention attribution baseline by explicitly separating early and late evidence and by respecting Swin’s window. Attention probabilities are collected from selected early blocks and selected late blocks via hooks placed at the point where attention probabilities are available. Then, gradients are obtained with respect to the target class score. For each chosen block, a gradient-weighted attention relevance matrix is computed and reduced to token importances via incoming-attention mass. These token importances are reshaped to window grids and then mapped back to the block’s spatial feature grid through a window-reversal operation that reconstructs the full feature map from its window partitions. When a block uses shifted windows, the reconstruction is corrected by undoing the cyclic shift so that all maps align in a common coordinate frame. The method then forms an early attention map by averaging the reconstructed maps from the first selected blocks and a late attention map by averaging those from the last selected blocks. The final EL-GWAA heatmap is a weighted linear combination of early and late maps [22].

#### 2.3.3. Token Feature Similarity

Token feature similarity (TFS) treats token embeddings as feature descriptors and computes per-token saliency from similarity relationships in embedding space [23]. This method assumes that tokens diverging from a global representation, or that display strong contrast relative to the overall context, are more likely to correspond to discriminative regions [24].

TFS maps are computed by comparing each token embedding to a global feature vector formed by pooling token embeddings over the full image. Similarity is measured with cosine similarity and converted into a spatial heatmap by reshaping tokens into a two-dimensional grid and upsampling to the input resolution. Hooked features are taken from normalization outputs within selected transformer blocks, which are readily available and represent stable token descriptors.

We propose two TFS variants in this work. In the token contrast map (TRAST) vs1, token feature similarity maps are computed across multiple depths and explicitly fused across early and late representations. A fixed number of early blocks and late blocks are selected, token descriptors are extracted from each, and a token-similarity map is computed per block. Early maps are averaged to obtain a low-level similarity map, and late maps are averaged to obtain a high-level similarity map. These two maps are then fused by a weighted linear combination, with a greater emphasis on late blocks to reflect their closer proximity to the classifier. The fused map is finally min–max normalized to yield a single TRAST heatmap per image.

TRAST vs2 retains the same similarity computation but changes the treatment of depth by keeping early and late maps explicitly separate rather than collapsing them into a single fused map. After generating independently normalized early and late similarity maps, the visualization overlays them in a layered representation in which late evidence can dominate where both signals overlap, while early evidence remains visible where late evidence is weak.

TRAST differs from attention rollout and transformer attention attribution because it does not propagate attention weights or use attention matrices as the heatmap. It also differs from Grad-CAM-family approaches because it does not require class gradients. TRAST computes the cosine similarity between each local token and the global token descriptor of the same image, inverts this similarity to emphasize locally distinctive features, normalizes the resulting token scores, and reconstructs a spatial map.

A detailed mathematical description and stepwise implementation of the TRAST algorithm are provided in Supplementary Tables S6–S8, the Supplementary Mathematical Definition and Justification of the Token contRAST (TRAST) Method, and Supplementary Algorithm S1.

Two variants of the proposed method (TRAST vs1 and TRAST vs2) were evaluated in Phase 1. Based on the screening results, TRAST vs1 was selected for Phase 2 evaluation and is referred to as “TRAST” in the subsequent analysis.

#### 2.3.4. Hybrid

The hybrid family of XAI methods is instantiated as a cosine-grad fusion map (CGFM), which fuses a gradient-based localization map (CAM-style, class-conditional) with a token-similarity map (global context, representation-driven). The motivation is that CAM maps can be sharply discriminative but may miss globally consistent context, whereas token-similarity maps can highlight broad regions of representational contrast but may be less tightly tied to the final decision boundary. CGFM therefore enforces agreement between the two by combining them into a single map or by presenting them jointly in a layered view.

CGFM vs1 computes the two constituent maps separately. First, a token-similarity map obtained by cosine similarity between late token descriptors and a global pooled descriptor, inverted and normalized. Secondly, a Grad-CAM map was computed on the final convolutional feature map for the same target class. The variant is primarily a presentation variant because it returns both maps and visualizes them as distinct layers. This variant is useful for diagnosing cases where the two signals disagree.

CGFM vs2 performs an explicit fusion of the two maps into a single heatmap. The default fusion strategy is an elementwise product, which emphasizes regions that are simultaneously salient under both signals and suppresses regions highlighted by only one branch. This yields a conservative explanation that prioritizes intersectional evidence, often producing sharper localization than token similarity alone while reducing the risk of CAM-only artifacts that lack contextual support. The fused map is then normalized to the unit interval for visual comparability.

CGFM-weighted generalizes the fusion by introducing continuous weights and alternative combination rules. method forms a weighted linear interpolation between the token-similarity map and the Grad-CAM map, followed by normalization. This variant provides a controlled trade-off between global context and local discriminative evidence and is used as the baseline hybrid.

### 2.4. Evaluation System

The evaluation system followed a two-phase design. In the first phase, candidate heatmap-generation methods were screened prior to clinician evaluation. This step aimed to remove methods that produced weak, artifactual, or anatomically implausible visualizations. In the second phase, the best-performing methods from Phase 1 were assessed by ophthalmologists through structured questionnaires focused on clinical plausibility.

#### 2.4.1. Participants

Phase 1 involved two expert reviewers: one ophthalmologist, who was not involved in the development of the explainability methods and was unaware of their implementation details, and one AI specialist who was part of the research team. Both reviewers independently evaluated the heatmaps.

Phase 2 involved 21 ophthalmologists, including 7 retina specialists, 7 general ophthalmologists, and 7 ophthalmology residents. Retina specialists and general ophthalmologists included clinicians with a range of experience (from >5 to >15 years), while the resident group comprised both senior and junior trainees. A supplementary evaluation form with additional image sets was completed by 4 participants (1 retina specialist with >5 years of experience, 1 general ophthalmologist with >5 years of experience, and 2 senior ophthalmology residents).

#### 2.4.2. Questionnaire

The Phase 2 questionnaire was designed to assess the clinical plausibility of the selected heatmaps. In each image set, participants were shown the original OCT image together with three XAI visualizations, corresponding to CGFM, Grad-CAM++, and TRAST. For each technique, respondents rated the degree to which the highlighted regions were consistent with the diagnosis assigned by the AI model, using a five-point Likert scale ranging from no agreement to very high agreement. The main form contained 10 image sets, and the supplementary form contained 20 additional image sets.

Images were randomly selected to represent all six diagnostic classes and a range of visual presentations and complexity levels within each class. Participants were unaware that a novel method was being evaluated. The order of images and corresponding heatmaps was randomized, and no grouping or ordering was applied based on expected rating.

#### 2.4.3. Statistical Assessment

Statistical analysis focused on Phase 2 questionnaire responses. Ratings were collected on a five-point Likert scale coded from 0 to 4 and were analyzed as ordinal data. For each explainability method, descriptive summaries included the mean, median, interquartile range (IQR), and the proportion of favorable ratings, defined as scores of 3 or 4.

For the main image-evaluation form and the supplementary image-evaluation form, method-level comparisons were performed using repeated-measures analyses, since the same participants evaluated all three explainability methods within each image set. For the overall comparison of the three methods within each form, participant-level summary scores were computed for each method and compared using the Friedman test. Effect size was quantified with Kendall’s coefficient of concordance (W). When the omnibus Friedman test was significant, post hoc pairwise comparisons between methods were performed using the Wilcoxon signed-rank test with Holm correction for multiple comparisons.

In addition to the overall participant-level analysis, image-level comparisons were carried out separately for each image set. The three method-specific ratings provided by the same participants were compared using the Friedman test in order to assess whether clinically perceived plausibility differed among methods within that specific case. Descriptive statistics were also computed across all image-level ratings within each form to summarize the overall rating distribution of each method.

To provide an integrated view of performance, ratings from the main and supplementary forms were also pooled and summarized descriptively across all available observations. These combined summaries included central tendency measures and the proportions of favorable ratings (3–4) and low ratings (0–1) for each method.

Additional stratified descriptive analyses were performed by diagnosis category and by participant subgroup. For diagnosis-specific analysis, ratings were pooled within each retinal disease category and summarized separately for each explainability method. Differences among professional groups, namely retina specialists, general ophthalmologists or other non-retina subspecialists, and ophthalmology residents, were assessed in the main form using respondent-level average scores for each method and the Kruskal–Wallis test.

All statistical tests were two-sided, and a significance threshold of *p* < 0.05 was used. Statistical analyses were performed using standard scientific computing libraries.

## 3. Results

### 3.1. Classification Results

We first report the diagnostic performance of the Swin Transformer in distinguishing among five retinal diseases and normal OCT cases.

At the 10-fold stratified cross-validation procedure performed using the combined dataset, Swin achieved an overall accuracy of 97% (95% CI: 96.5–97.5%), and AUC 0.98 (95% CI: 0.977–0.983) with confidence intervals (CIs) computed across folds.

The trained model was subsequently evaluated on the external dataset. We used resampling with replacement (*n* = 5000) to compute the CIs of the performance metrics. In the external dataset, accuracy dropped to 91.82% (95% CI: 88.56–94.77), and the corresponding AUC was 0.993 (95% CI: 0.986–0.997).

Class-wise diagnostic performance was strong overall. For CNV, Swin achieved a sensitivity of 98.2% (95% CI: 94–100) and a specificity of 92.4% (95% CI: 88.8–95.5). For CSR, Swin had a sensitivity of 78.0% (95% CI: 65.5–89.7) with an almost perfect specificity of 99.8% (95% CI: 99.5–99.9). For DME, the model reached 94.0% sensitivity (95% CI: 86.9–100) and 99.2% specificity (95% CI: 98–100). For DRUSEN, the model achieved 88.5% sensitivity (95% CI: 79.2–96.2) and 98.8% specificity (95% CI: 97.3–100). For MH, Swin reached 91.5% sensitivity (95% CI: 82.9–98.1) and 99.8% specificity (95% CI: 99.5–100). Finally, for the Normal class, the model achieved 98.0% sensitivity (95% CI: 93.9–100) and 99.6% specificity (95% CI: 98.8–100).

The external AUC was computed from softmax probability scores using a one-vs-rest multiclass procedure with bootstrap confidence intervals, whereas accuracy was computed from the final argmax class label. These metrics therefore capture different operating characteristics. AUC is threshold-independent and reflects the ability of the model to rank the true class above alternative classes across decision thresholds, while accuracy reflects the single selected class at the chosen operating point. The external dataset was approximately balanced across diagnostic categories. Therefore, the higher AUC despite lower external accuracy reflects probability-ranking performance with residual class-specific argmax errors, particularly in classes with lower sensitivity, such as CSR and DRUSEN.

Because heatmap-based explainability is most informative when derived from a well-performing model, the strong performance of the Swin model provides a reasonable basis for evaluating the clinical plausibility of its explanations. However, high diagnostic accuracy does not guarantee that the generated explanations are faithful to the model’s internal reasoning, and this distinction is considered in the interpretation of results.

### 3.2. Phase 1: Ranking of the XAI Algorithms and Exclusion of Low-Performing Ones

**Figure 2:**
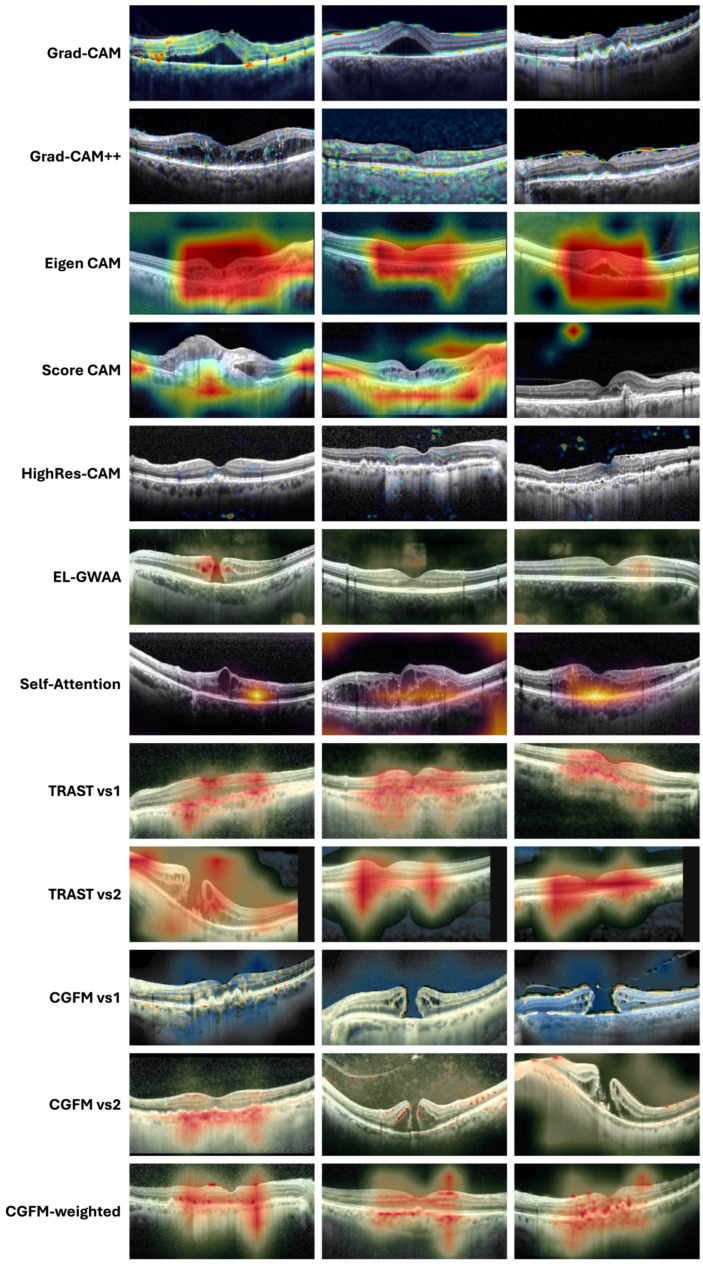
Representative heatmaps generated by each explainability method from the external evaluation dataset. Each row corresponds to a different XAI method (as labeled), while each column represents a different OCT image case. Heatmaps are overlaid on the original OCT B-scans to allow visual comparison of localization patterns across methods.

One ophthalmologist and one AI specialist independently evaluated 100 heatmaps per method from the external dataset (Figure 2). Heatmaps were scored using a five-point Likert scale (0–4). The complete Phase 1 scoring rubric is provided in Supplementary Table S3. Scores were based on visual quality, specifically considering (i) the presence of color OCT artifacts and (ii) highlighting of non-anatomical regions of the image. Reviewers were instructed to assign lower scores when heatmaps extended outside retinal layers, highlighted background or non-OCT regions, or showed diffuse or non-specific activation patterns without clear anatomical localization. The mean scores are presented in Table 4.

Statistical analysis showed a strong positive correlation between the ophthalmologist and the AI specialist. Based on the method-level mean scores, Kendall tau-b was 0.576 (*p* = 0.0088), and Spearman rho was 0.755 (*p* = 0.0045), indicating concordant ranking of the candidate methods.

Both experts consistently identified the same high- and low-performing methods, suggesting general agreement in the ranking of methods between the two evaluators. Based on mean scores, the three highest-rated methods were TRAST vs1, CGFM-weighted, and Grad-CAM++ (tied with CGFM vs2), which consistently outperformed the other XAI variants in providing anatomically accurate and artifact-free explanations.

Methods such as Eigen-CAM, Score-CAM, and some hybrid or attention-based variants often appeared diffuse or extended beyond the most relevant retinal structures, while Grad-CAM-based approaches were generally sharper but could still contain scattered irrelevant activations (Figure 2). By contrast, TRAST vs1 more often produced broader but anatomically centered responses, whereas TRAST vs2 appeared visually more diffuse. Figure 2 illustrates random heatmap examples from the external test set.

### 3.3. Phase 2: Analysis of the Specialists’ Responses on the XAI Forms

#### 3.3.1. Main Image-Evaluation Form

**Figure 3:**
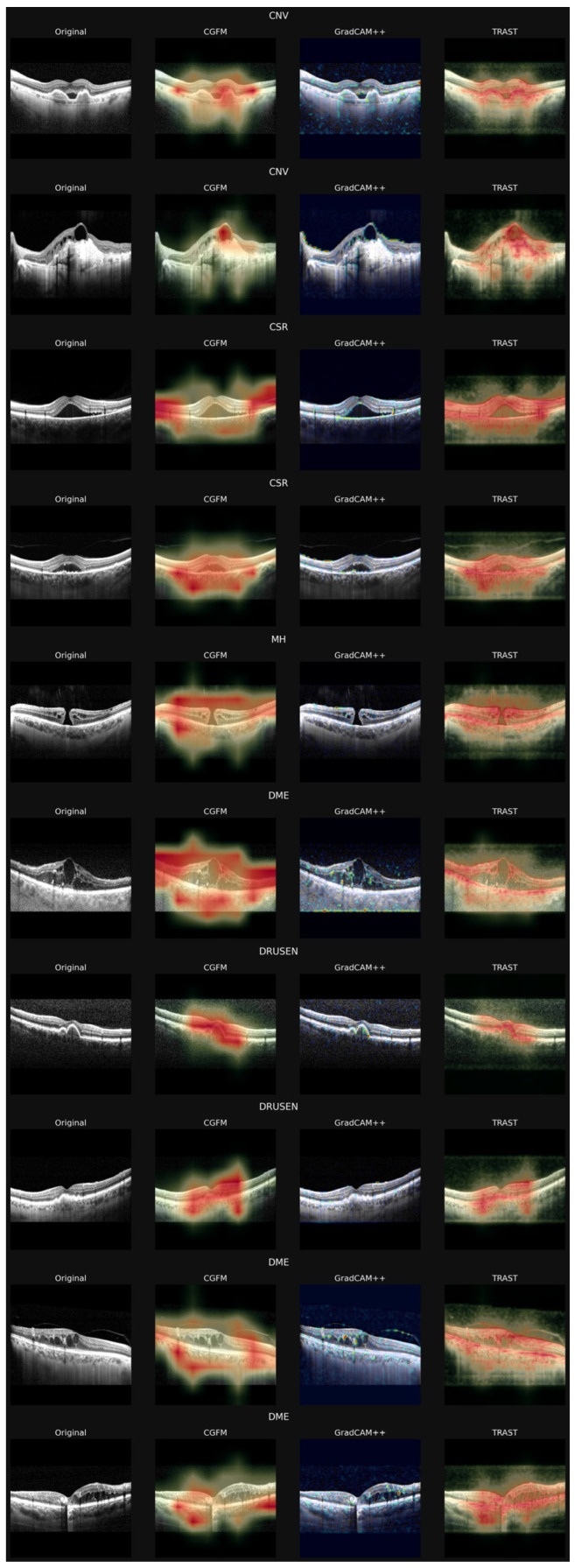
Main image-evaluation form used in the expert assessment process.

The main evaluation form included 21 complete responses, equally distributed among retina specialists (*n* = 7), general ophthalmologists or other non-retina subspecialists (*n* = 7), and ophthalmology residents (*n* = 7). A subset of four participants (1 retina specialist, 1 general ophthalmologist, and 2 residents) additionally completed the supplementary form with extended image sets. No missing data were observed. Figure 3 presents ten random panels given to the specialists.

In the main form, which contained 10 image sets and therefore 630 image-level technique ratings, TRAST achieved the highest ratings across all summary metrics. Its mean score was 2.49 with a median of 3 and an interquartile range of 2 to 3, and 61.0% of its ratings fell in the favorable 3 to 4 range. Grad-CAM++ was intermediate at a mean of 1.67, median of 2, and IQR of 1 to 2, with 24.3% favorable ratings. CGFM was lowest at a mean of 1.41, median of 1, and IQR of 0 to 2, with 20.5% favorable ratings. These results are summarized in Table 5.

At the respondent-summary level, the three techniques differed significantly overall by the Friedman test, chi-square = 14.34, *p* = 0.00077, with Kendall’s W = 0.34, which is a moderate repeated-measures effect. Post hoc Wilcoxon tests showed that TRAST was superior to CGFM after Holm correction, adjusted *p* = 0.0006, and also superior to Grad-CAM++, adjusted *p* = 0.010. CGFM versus Grad-CAM++ was only borderline, *p* = 0.051, so the real separation in the main form is mainly between TRAST and the other two techniques rather than between CGFM and Grad-CAM++.

The image-by-image pattern in the main form was consistent across cases, with TRAST achieving the highest or joint-highest ratings in the majority of image sets. The detailed image-level results are summarized in Table 6.

**Figure 4:**
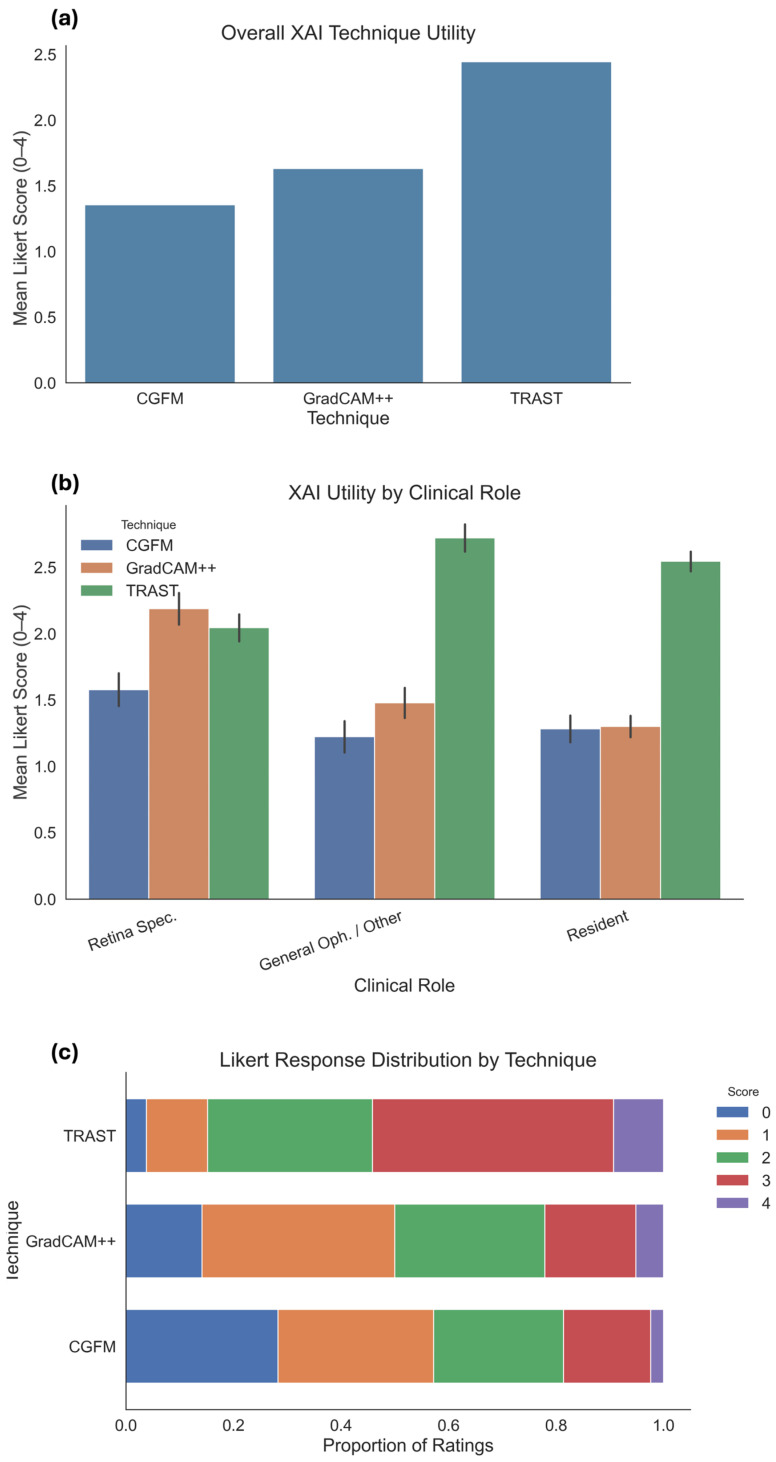
Statistical plots based on specialists’ responses as follows: (**a**) mean Likert score across the 21 specialists for each of the XAI variant of Phase 2; (**b**) mean Likert score between retina specialists, general ophthalmologists, and residents for each of the XAI variant of Phase 2; (**c**) proportion of ratings in the Likert scale assigned by the specialists to each XAI variant of Phase 2.

The overall statistical pattern is also reflected in Figure 4. Figure 4a shows the clear separation in mean Likert score between the three methods, with TRAST highest, Grad-CAM++ intermediate, and CGFM lowest. These ranks are preserved across the three professional groups (Figure 4b), although retina specialists appear more favorable to Grad-CAM++ than the other groups. Figure 4c further supports the same conclusion by showing that TRAST accumulated a much larger share of ratings in the higher part of the scale, whereas CGFM and Grad-CAM++ had a greater concentration of low ratings.

#### 3.3.2. Supplementary Image-Evaluation Form

The extra-images form adds 20 more image sets and 240 more ratings. The supplementary results showed a consistent descriptive trend favoring TRAST across most image sets. The descriptive pattern is again favorable to TRAST. Across all supplementary images, TRAST had a mean of 2.33, median of 2, and IQR of 2 to 3, with 36.3% favorable ratings of 3 to 4. Grad-CAM++ had a mean of 1.53 and median of 1, with 16.3% favorable ratings, while CGFM had a mean of 1.20 and median of 1, with 13.8% favorable ratings. A respondent-level Friedman test still reached significance, chi-square = 6.50, *p* = 0.039, with Kendall’s W = 0.81, but that very large effect size should not be overinterpreted because it is driven by only four raters. Pairwise Wilcoxon tests were not significant. Therefore, the supplementary analysis is treated as supportive evidence for the descriptive pattern observed in the main form rather than as a standalone confirmatory result.

The CNV supplementary block, sets 11 to 14, was consistently favorable to TRAST. Set 11 showed TRAST at 2.00 while both CGFM and Grad-CAM++ were only 0.75, narrowly missing significance at *p* = 0.058. Set 12 showed TRAST at 2.50, Grad-CAM++ at 1.75, and CGFM at 0.50, *p* = 0.030. Set 13 again favored TRAST at 2.25 over CGFM at 1.25 and Grad-CAM++ at 0.50, *p* = 0.037. Set 14 repeated the same pattern, with TRAST at 2.00 and the other two both below 1, *p* = 0.023. The extra CNV images strengthen the evidence that TRAST is more convincing than the alternatives for CNV localization.

The CSR supplementary block, sets 15 to 18, also leaned toward TRAST, though with more variability. Set 15 showed TRAST at 2.50, but the difference was not significant, *p* = 0.424. Set 16 was stronger, with TRAST at 2.75, CGFM at 2.25, and Grad-CAM++ at 1.50, *p* = 0.037. Set 17 still favored TRAST at 2.00 over 1.75 for CGFM and 0.75 for Grad-CAM++, *p* = 0.039. Set 18 gave the strongest CSR result in the supplementary block, with TRAST at 3.00, CGFM at 2.25, and Grad-CAM++ at 0.75, although the *p*-value remained 0.092 because of the small sample. In practical terms, the CSR extra images suggest that TRAST is again the most stable option, while CGFM can sometimes perform reasonably well, and Grad-CAM++ is the least consistent.

The DME supplementary block, sets 19 to 22, is the one area where Grad-CAM++ most clearly challenges TRAST. Set 19 favored TRAST at 2.50 over Grad-CAM++ at 1.75 and CGFM at 1.00, *p* = 0.092. Set 20 was one of the few images where Grad-CAM++ came first, with a mean of 2.25 versus 2.00 for TRAST and 0.50 for CGFM, *p* = 0.023. Set 21 repeated that pattern, with Grad-CAM++ at 2.50, TRAST at 2.00, and CGFM at 0.50, *p* = 0.061. Set 22 returned the advantage to TRAST at 2.50, while CGFM and Grad-CAM++ were both only 0.75, *p* = 0.085. So, for these extra DME images, TRAST is still competitive, but Grad-CAM++ appears much more image-sensitive and sometimes does better.

The DRUSEN supplementary block, sets 23 to 26, was more mixed and generally friendlier to both TRAST and CGFM than to Grad-CAM++. Set 23 was actually the strongest Grad-CAM++ case in the entire supplementary form, with a mean of 3.25, ahead of TRAST at 2.75 and CGFM at 2.25, although the *p*-value was only 0.156. Set 24 strongly favored TRAST at 2.75, with CGFM at 2.25 and Grad-CAM++ at 0.75, *p* = 0.024. Set 25 again had TRAST highest at 2.75, but CGFM was close at 2.50, and Grad-CAM++ lagged at 1.25, *p* = 0.097. Set 26 was nearly a tie between TRAST and CGFM, both at 2.25, with Grad-CAM++ at 1.50, *p* = 0.584. This disease category therefore looks less one-sided than CNV or MH, but even here TRAST remains at least competitive in every image and clearly better than Grad-CAM++ in most of them.

The MH supplementary block, sets 27 to 30, was notable because CGFM essentially collapsed. Its means ranged only from 0.25 to 0.50 across those four images. TRAST and Grad-CAM++ were much stronger. Set 27 still favored TRAST slightly, 1.50 versus 1.25 for Grad-CAM++ and 0.50 for CGFM, *p* = 0.097. Sets 28 and 29 showed Grad-CAM++ at 2.25, tying or slightly exceeding TRAST at 2.25 and 2.00. Set 30 gave Grad-CAM++ its clearest MH advantage at 2.50 versus 2.25 for TRAST and 0.25 for CGFM, *p* = 0.058. So, for the extra MH images, the practical conclusion is that CGFM is not convincing, while TRAST and Grad-CAM++ are the methods that remain clinically plausible.

#### 3.3.3. Combined Pattern Across All Image Ratings

We merged the main and supplementary image ratings, ending up with 870 technique-specific observations. Overall, TRAST has a mean of 2.45, a median of 3, and 54.1% favorable ratings of 3 to 4. Grad-CAM++ has a mean of 1.63, a median of 1.5, and 22.1% favorable ratings. CGFM has a mean of 1.36, a median of 1, and 18.6% favorable ratings. 55.2% of TRAST ratings were 3 or 4, while only 22.1% of Grad-CAM++ and 18.6% of CGFM reached that range. At the low end, 57.2% of CGFM ratings and 50.0% of Grad-CAM++ ratings were 0 or 1, compared with only 15.2% for TRAST.

**Figure 5:**
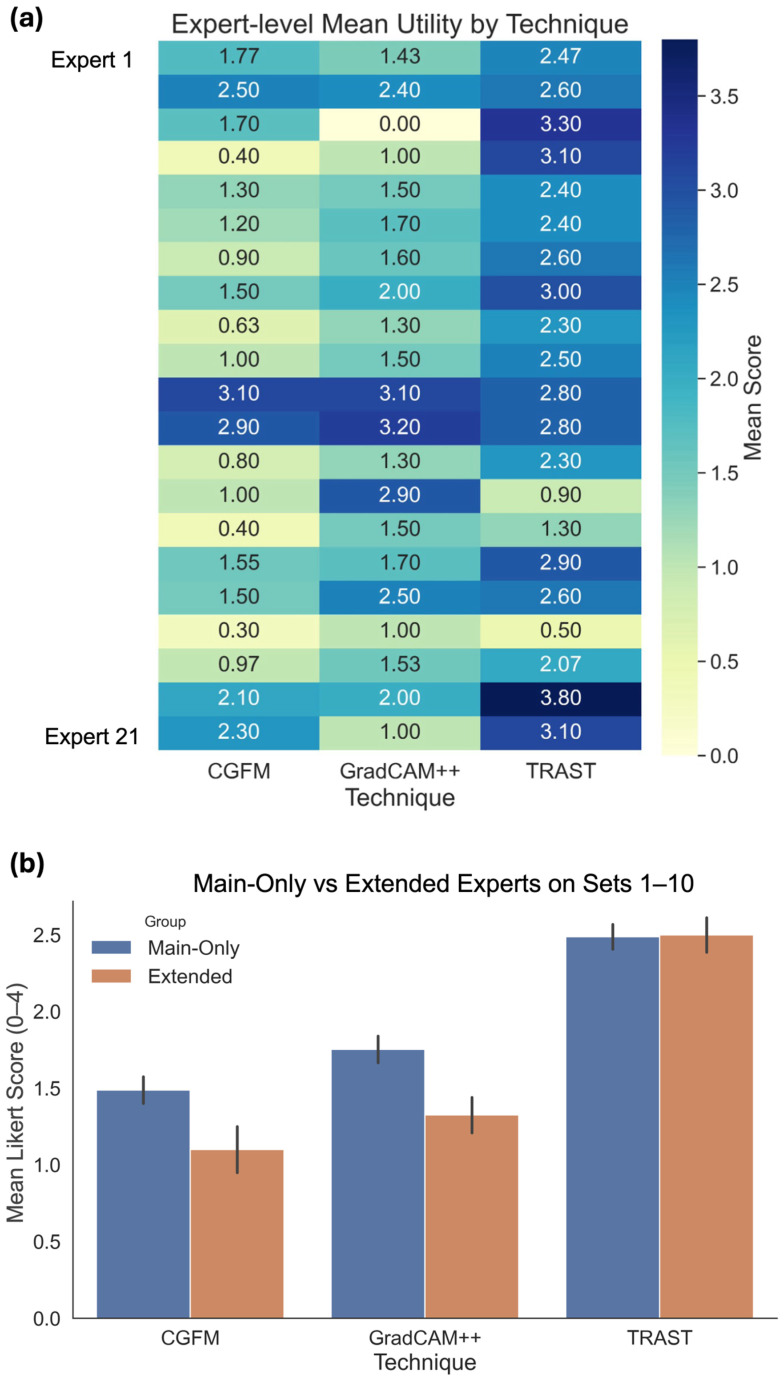
(**a**) Mean Likert-scale score values assigned by each specialist in Phase 2; (**b**) mean Likert-scale score of each XAI variant for the specialists who rated ten XAI image sets (main-only) and for the specialists who rated thirty XAI image sets (extended).

Figure 5 provides additional detail at the individual expert level. The heatmap in Figure 5a shows that most experts gave their highest mean scores to TRAST, although some heterogeneity is present, and a smaller number of raters evaluated Grad-CAM++ more favorably in selected cases. Figure 5b shows that the mean score of TRAST remained stable between the main-only raters and the extended raters, whereas CGFM and Grad-CAM++ were lower in the extended group.

#### 3.3.4. Differences by Diagnosis

When the ratings are pooled by diagnosis, TRAST remains the strongest method in every disease category. Its mean scores were 2.43 for CNV, 2.47 for CSR, 2.38 for DME, 2.62 for DRUSEN, and 2.33 for MH. DRUSEN and CSR were therefore the most favorable disease categories for TRAST, while MH was the hardest. Grad-CAM++ was relatively strongest in DME and MH, both around 1.83 to 1.85, and weakest in CNV at 1.28. CGFM was relatively strongest in DRUSEN at 1.98 and CSR at 1.64, but weak in DME at 0.90 and MH at 0.88. The largest within-diagnosis separation among techniques appeared in DME and MH, where the pooled Friedman effect sizes were strongest, suggesting that raters saw especially clear qualitative differences between the three explanation methods in those pathologies. Diagnosis-specific formal test results are provided in Supplementary Table S4, and post hoc diagnosis-specific Wilcoxon-Holm comparisons are reported in Supplementary Table S5.

#### 3.3.5. Differences by Professional Group

In the main form, there was no statistically significant difference among retina specialists, general ophthalmologists, or other subspecialists, and residents in their respondent-level average scores for any of the three techniques. Kruskal–Wallis *p*-values were 0.327 for CGFM, 0.126 for Grad-CAM++, and 0.357 for TRAST. Retina specialists tended to score Grad-CAM++ higher on average than the other groups, while residents and general ophthalmologists had very similar central tendencies for TRAST. Even so, the subgroup spreads were not large enough to support a stable group effect, and the safest interpretation is that the broad preference for TRAST generalizes reasonably well across experience levels. The supplementary form is too small for meaningful between-group analysis.

**Figure 6:**
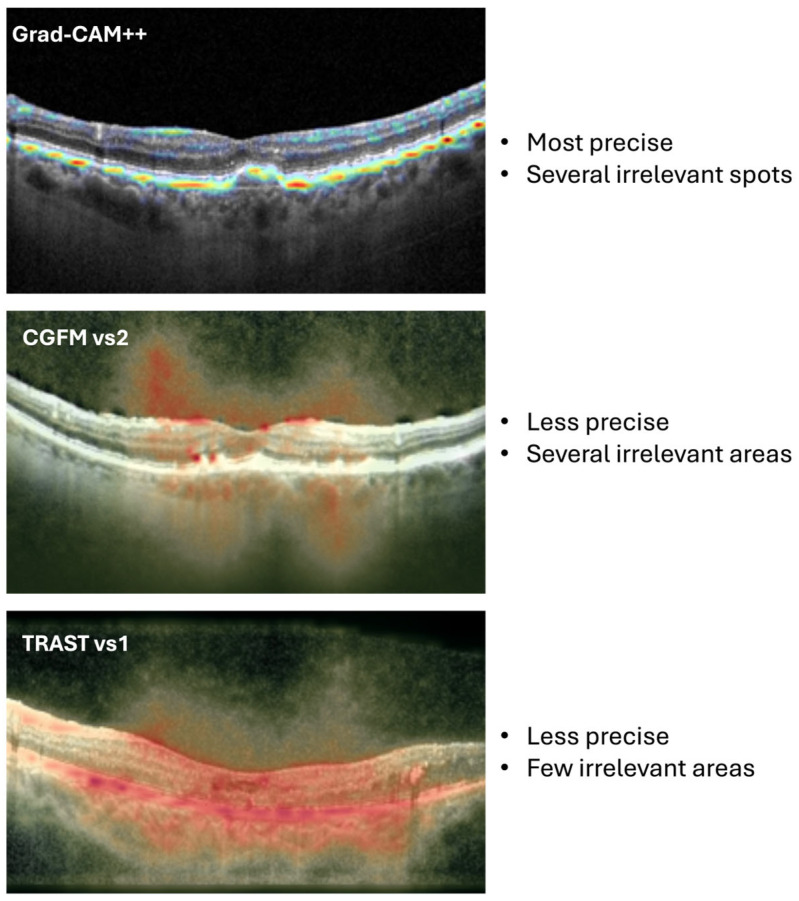
Comparison between the Grad-CAM++, CGFM (vs2), and TRAST (vs1) visual representations.

The direct visual comparison in Figure 6 clarifies the practical difference between the three methods. Grad-CAM++ appears to be the most spatially precise method, but this precision is accompanied by several irrelevant focal activations. CGFM vs2 is less precise and also includes broader irrelevant areas. TRAST vs1 is less sharply localized than Grad-CAM++, but it shows fewer irrelevant areas and a more anatomically coherent distribution of relevance. This comparison explains why experts may have preferred TRAST overall. In clinical interpretation, slightly broader but anatomically plausible highlighting may be judged more useful than very sharp maps that also contain distracting or implausible activations.

## 4. Discussion

AI-based models in retinal imaging have demonstrated high diagnostic performance; however, successful clinical translation requires more than predictive accuracy. Clinicians need explanations that are interpretable, clinically meaningful, and aligned with real-world decision-making tasks. Despite this, many prior studies focus primarily on performance metrics and either omit explainability or provide only limited qualitative examples without structured evaluation [25,26,27,28]. This aligns with recent work in medical XAI, where visually plausible explanations are commonly presented, but systematic multi-expert evaluation remains limited.

In this study, multiple explainability-enhancing algorithms were evaluated for their ability to highlight diagnostically relevant regions in OCT images for multi-class retinal disease classification using a Swin transformer network. A two-phase evaluation framework was employed, combining initial technical screening with structured multi-expert clinical assessment. This design provides a practical approach for selecting explainability methods when multiple alternatives are available.

Among the evaluated methods, TRAST, which was first introduced in this study, received the highest clinical plausibility ratings from specialists. Grad-CAM++ showed intermediate performance and was competitive in specific cases, particularly in DME and MH images, whereas CGFM was the weakest overall. These findings should be interpreted in the context of the strong classification performance of the Swin model, as explainability assessment is most meaningful when applied to a well-performing classifier with both internal and external evaluation. High diagnostic performance was considered a prerequisite for meaningful heatmap evaluation, because explanations of a poorly performing classifier may reflect unstable or incorrect decision rules. However, high accuracy does not guarantee explanation fidelity. If the same framework is applied to models with more modest performance, or to models with systematic errors in specific disease classes, heatmaps should be evaluated separately for correct and incorrect predictions and stratified by diagnosis category.

Importantly, specialist evaluations suggest that clinical preference is not driven solely by spatial sharpness. TRAST, although less sharply localized than Grad-CAM++, was often preferred because it produced broader but more anatomically coherent maps, with fewer clearly irrelevant activations. Grad-CAM++ was visually sharper but more likely to contain scattered highlights outside the most plausible retinal regions. CGFM, especially in the weaker-performing variants, appeared less stable and less clinically convincing. In practice, the most useful explanation may not be the one with the finest boundaries, but the one whose overall distribution of relevance is more consistent with retinal morphology and disease-related structure. This observation is consistent with the visual comparisons (Figure 6) and the overall rating distributions (Figure 4 and Figure 5). This pattern should not be interpreted as evidence that broader heatmaps are inherently more faithful or more precise. Rather, it indicates that, in a clinical plausibility setting, scattered focal activations in anatomically implausible regions may reduce perceived usefulness even when spatial localization appears sharp. Smoother or more visually coherent heatmaps may appear more convincing to clinicians even when their relationship to the model’s internal reasoning remains uncertain. Quantifying the trade-off between spatial precision, anatomical plausibility, and faithfulness requires future comparison with expert annotations and perturbation-based validation.

An important limitation of the study is that the questionnaire assessed perceived compatibility with the diagnosis, which is a judgment of plausibility, but not a direct measure of faithfulness to the model’s internal reasoning. The Phase 2 evaluation, therefore, reflects clinical plausibility rather than causal model interpretability, as it did not include perturbation testing, feature removal, or intra-model consistency analysis. Therefore, the present findings should be interpreted strictly as a structured assessment of clinical plausibility and not as evidence of causal model interpretability. Clinician preference for a particular heatmap should therefore not be interpreted as confirmation that the highlighted regions faithfully represent the model’s internal decision process. In addition, the study focused on a single classification backbone and on a selected set of heatmap families, so the conclusions should not yet be generalized to all OCT models or all explanation methods. Although the present empirical validation was limited to a Swin Transformer applied to multiclass OCT retinal disease classification, the proposed two-phase evaluation framework is conceptually model-agnostic and may be applicable to other architectures, imaging modalities, and clinical tasks, subject to further validation in these settings.

The combination of public and private datasets may introduce distributional bias, particularly given the numerical imbalance between the large public dataset and the smaller private dataset, despite the stratified manner of the training procedure. As a result, the contribution of the private dataset to model training might be limited relative to the public data. Nevertheless, the private dataset may still contribute by increasing exposure to image heterogeneity and acquisition variability during training, potentially providing a useful regularization signal despite its smaller size. However, the inclusion of an independent external dataset for evaluation partially mitigates this concern by providing a more realistic assessment of generalization across data sources.

An additional limitation concerns the evaluation design in Phase 1. The inclusion of an AI specialist from the research team may introduce a potential source of bias; however, the ophthalmologist reviewer was not involved in the development of the explainability methods and was unaware of the presence of a newly proposed method, which supports the objectivity of the evaluation. Furthermore, the use of only two raters and the absence of formal blinding may limit the objectivity of this preliminary screening step. However, Phase 1 was intended solely for initial quality control, and the primary comparative evaluation was conducted in Phase 2 using a larger group of independent clinicians.

Another limitation is that this study does not include a comparison with objective interpretability metrics or a direct evaluation of how explainability maps influence clinical decision-making. Broader aspects of clinician interaction with XAI, including understanding, trust, and perceived decision support, were collected and will be reported in a separate study.

Future work should include larger multicenter studies with prospectively defined datasets and a greater number of expert raters. Combining clinician-based evaluation with objective technical validation, such as comparison with expert annotations or perturbation-based faithfulness tests, and evaluating the impact of explainability maps on clinical decision-making would provide a more comprehensive assessment of explainability methods. Furthermore, it remains important to investigate whether the use of explainability tools influences clinician performance, confidence calibration, and decision-making in real-world settings. Such work would move the field from plausibility assessment toward clinically consequential evidence. These directions align with current reporting and evaluation frameworks for medical AI, including MI-CLAIM [29], CLAIM [30,31], DECIDE-AI [32], SPIRIT-AI [33], CONSORT-AI [33], and FUTURE-AI [34], which emphasize transparency, clinical plausibility, and human-centered evaluation.

## 5. Conclusions

This study presents a structured two-phase framework for evaluating explainability methods in transformer-based OCT classification using multi-expert clinical assessment. Among the evaluated methods, the proposed TRAST demonstrated the highest clinical plausibility, consistently outperforming Grad-CAM++ and CGFM across multiple evaluation settings. These results reflect perceived clinical plausibility rather than direct model interpretability, highlighting the importance of combining human-centered evaluation with quantitative approaches. In this context, structured human assessment provides a systematic and clinically meaningful way to evaluate explanation maps alongside conventional metrics.

Building on these findings, clinically useful explanations are not necessarily those with the highest spatial precision, but those that align with anatomical structure and disease-relevant features. This work supports the integration of structured human evaluation as a complementary component in the assessment of explainability methods. While the present results are based on a single model and dataset configuration, the proposed framework remains conceptually model-agnostic and may extend to other architectures, imaging modalities, and clinical tasks, subject to further validation in these settings. Incorporating structured human evaluation into validation pipelines, alongside quantitative methods and external datasets, may improve the reliability and clinical applicability of artificial intelligence systems in ophthalmology.

## Figures and Tables

**Table 1:** Class distribution across datasets.

Class	OCT-C8	GEN	OMM
CNV	3000	59	56
DRUSEN	3000	57	52
DME	3000	53	51
CSR	3000	47	46
MH	3000	49	47
NORMAL	3000	69	54
**Total**	**18,000**	**334**	**306**

CNV (choroidal neovascularization); CSR (central serous chorioretinopathy); DME (diabetic macular edema); MH (macular hole).

**Table 2:** Training parameters of the Swin Transformer model.

Parameter	Value
Backbone	Timm swinv2_base_window8_256.ms_in1k with ImageNet-pretrained weights
Input	256 × 256 × 3
Classifier head	Linear (embed_dim, 512), ReLU, Dropout 0.5, Linear (512, 6)
Trainable layers	Freeze_backbone = True and unfreeze_last_n_stages = 20; for SwinV2-base, this unfreezes all stages, and final normalization layers are trainable
Loss	FocalWithLS enabled: focal gamma = 2.0 and label smoothing = 0.05
Optimizer	AdamW, weight decay = 1 × 10^−4^, default PyTorch betas and epsilon
Learning rates	Two parameter groups: backbone learning rate = 3 × 10^−5^; classifier-head learning rate = 3 × 10^−4^
Scheduler	ReduceLROnPlateau, monitored validation accuracy, mode = max
Scheduler parameters	CV: factor 0.5, patience 3; full model: factor 0.5, patience 4; external/special protocol: factor 0.8, patience 5
Epochs	Maximum 100 epochs
Early stopping	Minimum 10 epochs before stopping; CV patience 10, full-model patience 10, standard external-validation patience 8, special external protocol patience 12
Batch size	16 for standard CV/full training; 32 in the special external-validation protocol
Sampling	WeightedRandomSampler used when class_balanced = True in external-validation/special protocol
Main random seed	56 for training; 42 for XAI panel generation
Software/hardware	Python 3.11.14, PyTorch 2.5.1, torchvision 0.20.1, timm 1.0.22, CUDA 12.1, NVIDIA GeForce RTX 3080 Ti 12 GB

ReLU (rectified linear unit); CUDA (compute unified device architecture); CV (cross-validation); XAI (explainable artificial intelligence).

**Table 3:** Explainability heatmap algorithms.

Family	Method	Variant
CAM	Grad-CAM	Baseline
CAM	Grad-CAM++	Baseline
CAM	Score-CAM	Baseline
CAM	Eigen-CAM	Baseline
CAM	HighRes-CAM	Baseline
TAA	EL-GWAA	Baseline
TAA	Self-Attention	Baseline
TFS	TRAST	vs1
TFS	TRAST	vs2
Hybrid	CGFM	vs1
Hybrid	CGFM	vs2
Hybrid	CGFM-weighted	Baseline

CAM (class activation mapping); CGFM (cosine-grad fusion map); EL-GWAA (early–late gradient-weighted window attention aggregation); Grad-CAM (gradient-weighted class activation mapping); TAA (transformer attention attribution); TFS (token feature similarity); TRAST (token contrast map).

**Table 4:** Phase 1 mean scores.

Method	Variant	Ophthalmologist	AI-Specialist	Mean Score
Grad-CAM	Baseline	2.26	2.88	2.57
Grad-CAM++	Baseline	3.14	2.94	3.04
Score-CAM	Baseline	1.55	1.03	1.29
Eigen-CAM	Baseline	1.47	1.17	1.32
HighRes-CAM	Baseline	2.24	1.88	2.06
EL-GWAA	Baseline	2.45	2.13	2.29
Self-Attention	Baseline	2.35	3.17	2.76
TRAST	vs1	3.38	3.25	3.32
TRAST	vs2	2.1	2.56	2.33
CGFM	vs1	2.72	2.41	2.57
CGFM	vs2	3.19	2.89	3.04
CGFM-weighted	Baseline	3.03	3.36	3.2

CAM (class activation mapping); CGFM (cosine-grad fusion map); EL-GWAA (early–late gradient-weighted window attention aggregation); Grad-CAM (gradient-weighted class activation mapping); TRAST (token contrast map).

**Table 5:** Summary of ratings across explainability methods (main form).

Method	Mean	Median	IQR	Favorable Ratings (%)
TRAST	2.49	3	2–3	61.0%
Grad-CAM++	1.67	2	1–2	24.3%
CGFM	1.41	1	0–2	20.5%

CGFM (cosine-grad fusion map); Grad-CAM++ (gradient-weighted class activation mapping); IQR (interquartile range); TRAST (token contrast map).

**Table 6:** Mean clinician ratings per image set for each explainability method (main evaluation form).

Image Set	Diagnosis	TRAST	Grad-CAM++	CGFM	*p*-Value
1	CNV	2.48	1.52	1.71	0.005
2	CSR	2.14	2.14	1.00	0.006
3	DME	2.24	2.05	1.00	<0.001
4	DRUSEN	2.76	1.38	2.00	<0.001
5	MH	2.33	1.38	0.76	<0.001
6	CNV	2.57	1.33	1.48	<0.001
7	CSR	2.71	1.52	2.10	0.002
8	DME	2.62	1.67	0.95	<0.001
9	DRUSEN	2.48	1.62	1.71	0.007
10	MH	2.57	2.10	1.43	<0.001

CGFM (cosine-grad fusion map); CNV (choroidal neovascularization); CSR (central serous chorioretinopathy); DME (diabetic macular edema); Grad-CAM++ (gradient-weighted class activation mapping); MH (macular hole); TRAST (token contrast map). *p*-values correspond to Friedman tests assessing differences among methods within each image set.

## Data Availability

The public OCT-C8 dataset used in this study is openly available. The private datasets are not publicly available due to institutional and privacy restrictions, but may be available from the corresponding author upon reasonable request and with permission from the respective institutions.

## Supplementary Materials

The following supporting information can be downloaded at: https://www.mdpi.com/article/10.3390/jimaging12050211/s1. Table S1: Preprocessing and augmentation information; Table S2: XAI method implementation details; Table S3: Evaluation phase 1 scoring rubric; Table S4: Diagnosis-specific formal test results; Table S5: Post hoc diagnosis-specific Wilcoxon–Holm results; Table S6: Mathematical notation used in the TRAST definition; Table S7: TRAST implementation settings used for the OCT explainability experiments; Table S8: Algorithmic summary of TRAST for a single OCT B-scan; Mathematical Definition and Justification of the Token contRAST (TRAST) Method; Algorithm S1: Token contRAST (TRAST) heatmap generation for one OCT B-scan.

## Abbreviations

The following abbreviations are used in this manuscript:

AI	Artificial Intelligence
AMD	Age-Related Macular Degeneration
CAM	Class Activation Mapping
CGFM	Cosine-Grad Fusion Map
CIs	Confidence Intervals
CNV	Choroidal Neovascularization
CSR	Central Serous Chorioretinopathy
DME	Diabetic Macular Edema
EL-GWAA	Early–Late Gradient-Weighted Window Attention Aggregation
Grad-CAM	Gradient-Weighted Class Activation Mapping
MH	Macular Hole
OCT	Optical Coherence Tomography
RVO	Retinal Vein Occlusion
TAA	Transformer Attention Attribution
TFS	Token Feature Similarity
TRAST	Token contRAST Map
ViT	Vision Transformer
XAI	Explainable Artificial Intelligence
