# Supplementary material for: Clinician-Centered Evaluation Framework for Explainable AI Heatmaps in OCT-Based Retinal Disease Classification
Source: J Imaging. 2026 May 16;12(5):211. doi: 10.3390/jimaging12050211 (PMC13207473; doi:10.3390/jimaging12050211)
Supplement: Supplementary file 1 [file jimaging-12-00211-s001.zip › jimaging-4276109-supplementary.pdf]

**SUPPLEMENTARY MATERIAL**

# "Clinician-Centered Evaluation Framework for Explainable AI Heatmaps in OCT-Based Retinal Disease Classification"

**Eirini Maliagkani [1,*], Ilias Georgalas [1], Ioannis Datseris [2], Elpiniki Papageorgiou [3], and Ioannis D. Apostolopoulos [3]**

[1] 1st Department of Ophthalmology, General Hospital of Athens "G. Gennimatas", National and Kapodistrian University of Athens, Athens, Greece
[2] OMMA Ophthalmological Institute of Athens, Athens, Greece
[3] Artificial intelligence, Computational Methods and Technological Applications (ACTA) Lab, Department of Energy Systems, University of Thessaly, Gaiopolis Campus, Larisa, Greece

**Table S1.** Preprocessing and augmentation information.

| *Component* | *Implemented procedure* |
|---|---|
| Image loading | All images were read with orientation correction and converted to grayscale float arrays in [0,1]. 8-bit and 16-bit inputs were supported. |
| Resize | Aspect-ratio-preserving LANCZOS resize with letterbox padding to 256 x 256 pixels; no crop was applied. |
| Channels | The single grayscale channel was replicated to three channels for compatibility with ImageNet-pretrained SwinV2. |
| Contrast harmonization | CLAHE, clip limit 2.0, 8 x 8 tile grid. |
| Intensity normalization | Robust 2nd-98th percentile rescaling; optional scanner-invariant fixed-range rescaling and histogram matching using a reference histogram estimated from sampled images. |
| Input normalization | ImageNet mean [0.485, 0.456, 0.406] and standard deviation [0.229, 0.224, 0.225]. |
| Training augmentation | Horizontal flip p = 0.5; random rotation +/-15 degrees; affine translation 0.03/0.03 and scale 0.89-1.15; brightness/contrast jitter 0.3; random gamma 0.8-1.2 with p = 0.7; Gaussian blur kernel 3 with p = 0.05. |
| Validation preprocessing | No stochastic augmentation; tensor conversion and ImageNet normalization only. |
| External test-time augmentation | When enabled, 9 stochastic views per image: horizontal flip p = 0.5, affine translation 0.04/0.04, scale 0.90-1.10, brightness/contrast jitter 0.4, gamma 0.7-1.3 with p = 0.8, Gaussian blur p = 0.05. |

**Table S2.** XAI method implementation details.

| *Method/family* | *Layer, block, or implementation detail* |
|---|---|
| Grad-CAM, Grad-CAM++, HiRes-CAM | CAM_Variants target-layer search prioritizes the last Conv2d. In timm SwinV2-base this is patch_embed.proj, Conv2d(3,128,kernel=4,stride=4). |
| Eigen-CAM | CAM_eigen_score selects the final LayerNorm first; for SwinV2-base this is backbone.norm. |
| Score-CAM | Same final LayerNorm target as Eigen-CAM; channel computation restricted to the 64 channels with highest activation variance. |
| Self-attention attribution | Hook at backbone.layers[-1].blocks[-1].attn.softmax, i.e. layers.3.blocks.1.attn.softmax. |
| EL-GWAA / ELGAR | Hooks attention probabilities at attn.attn_drop input for layers.0.blocks.0, layers.0.blocks.1, layers.3.blocks.0, and layers.3.blocks.1; low/high fusion weights 0.4/0.6. |
| TRAST vs1 | Hooks norm2, with norm1/block fallback, in first two and last two Swin blocks; computes inverted cosine similarity to the global token descriptor; fuses early/late maps as 0.4 low + 0.6 high. |
| TRAST vs2 | Same maps as TRAST vs1 but displayed as two separate overlay layers rather than one fused map. |
| CGFM vs1 | Displays token-similarity and Grad-CAM maps as separate layers for the same predicted class. |
| CGFM vs2 | Uses elementwise product of the token-similarity map and Grad-CAM map, followed by normalization. |
| CGFM-weighted | Uses weighted sum or weighted geometric combination of token-similarity and Grad-CAM maps, default weights 0.5/0.5. |

**Table S3.** Evaluation phase 1 scoring rubric.

| *Score* | *Category* | *Criterion* |
|---|---|---|
| 0 | Unusable | Dominant artifacts or no plausible retinal localization |
| 1 | Poor | Mostly non-anatomical, misleading, or irrelevant highlighting |
| 2 | Fair | Some relevant retinal highlighting but substantial diffusion, noise, or irrelevant activation |
| 3 | Good | Most highlighted regions anatomically plausible with only minor artifacts or ambiguity |
| 4 | Excellent | Clear disease-relevant retinal localization with minimal irrelevant activation |

**Table S4**. Diagnosis-specific formal test results.

| *Diagnosis* | *Triplets* | *CGFM mean* | *Grad-CAM++ mean* | *TRAST mean* | *Friedman p* | *Kendall W* |
|---|---|---|---|---|---|---|
| CNV | 58 | 1.38 | 1.28 | 2.43 | 9.09e-13 | 0.478 |
| CSR | 58 | 1.64 | 1.66 | 2.47 | 3.66e-05 | 0.176 |
| DME | 58 | 0.90 | 1.84 | 2.38 | 8.63e-13 | 0.479 |
| DRUSEN | 58 | 1.98 | 1.55 | 2.62 | 1.24e-07 | 0.274 |
| MH | 58 | 0.88 | 1.83 | 2.33 | 6.64e-12 | 0.444 |

**Table S5.** Post hoc diagnosis-specific Wilcoxon-Holm results.

| *Diagnosis* | *Holm-adjusted p values* |
|---|---|
| CNV | TRAST vs CGFM: 1.65e-07; TRAST vs Grad-CAM++: 4.14e-07; Grad-CAM++ vs CGFM: 0.407 |
| CSR | TRAST vs CGFM: 3.18e-05; TRAST vs Grad-CAM++: 0.000978; Grad-CAM++ vs CGFM: 0.811 |
| DME | TRAST vs CGFM: 6.73e-09; TRAST vs Grad-CAM++: 0.00674; Grad-CAM++ vs CGFM: 5.21e-06 |
| DRUSEN | TRAST vs CGFM: 4.65e-05; TRAST vs Grad-CAM++: 3.04e-05; Grad-CAM++ vs CGFM: 0.0192 |
| MH | TRAST vs CGFM: 1.29e-07; TRAST vs Grad-CAM++: 0.0214; Grad-CAM++ vs CGFM: 8.11e-05 |

## Mathematical Definition and Justification of the Token contRAST (TRAST) Method

### 1.1. Purpose and Scope

TRAST is a token-feature contrast method for generating heatmaps from a trained Swin Transformer OCT classifier. The method uses the geometry of hidden token embeddings rather than gradients alone. For each selected transformer block, TRAST compares each spatial token with the global token descriptor of the same image. Tokens that deviate from the dominant global representation receive higher contrast scores and are displayed as more salient regions.

This supplement gives the formal definition, implementation details, and mathematical rationale for TRAST. The heatmap should be interpreted as a model-internal clinical plausibility map: it identifies image regions whose learned representations are locally distinctive within the same OCT B-scan. It is not presented as proof of a causal mechanism for the classifier decision.

- The method uses token descriptors captured from normalized Swin block outputs.

- Token saliency is defined by inverse cosine similarity to the block-level global descriptor.

- Each block map is min-max normalized, reshaped to its token grid, and bilinearly resized to the OCT input size.

- The final TRAST map fuses early and late Swin representations, with greater weight assigned to late diagnostic features.

### 1.2. Notation

**Table S6**. Mathematical notation used in the TRAST definition.

| **Symbol** | **Meaning** |
|---|---|
| x | Input OCT B-scan after preprocessing and resizing to H x W pixels. |
| f_theta | Trained Swin Transformer classifier with parameters theta. |
| y_hat | Predicted class of f_theta(x). |
| l | Index of a selected Swin transformer block. |
| Z_l | Token descriptor matrix captured from block l. |
| z_(l,i) | Descriptor of token i in block l. |
| N_l | Number of spatial tokens in block l. |
| d_l | Embedding dimension of block-l tokens. |
| g_l | Global block descriptor obtained by average pooling tokens. |
| c_(l,i) | Cosine similarity between token z_(l,i) and global descriptor g_l. |
| q_(l,i) | Token contrast score before normalization. |
| M_l | Block-level TRAST map resized to the OCT input dimensions. |
| E, L | Early-block and late-block sets. |
| M_E, M_L | Averaged early and late maps. |
| H_TRAST | Final TRAST heatmap. |

### 1.3. Token Descriptor Extraction

Let x be the preprocessed OCT B-scan and f_theta be the trained Swin Transformer classifier. During a forward pass, TRAST registers forward hooks on selected transformer blocks. In the implementation used in the study, the preferred hook is the block norm2 module, with norm1 or the block output used only as fallback if norm2 is unavailable. For block l, the captured token matrix is:

$$Z_l = [z_{l,1}, z_{l,2}, \dots, z_{l,N_l}] in\ R^{N_l x\ d_l} \quad \text{(S1)}$$

The implementation accepts either already flattened tokens with shape (B, N_l, d_l) or feature grids with shape (B, H_l, W_l, d_l) or (B, d_l, H_l, W_l), which are flattened to N_l = H_l W_l tokens before scoring.

### 1.4. Global Descriptor and Token Contrast

For each selected block, TRAST computes a global descriptor by averaging all tokens from the same image and the same layer:

$$g_l = \left(\frac{1}{N_l}\right) sum_{i=1}^{N_l} z_{l,i} \quad \text{(S2)}$$

Each token is then compared with this global descriptor by cosine similarity:

$$c_{l,i} = \frac{(z_{l,i}^T g_l)}{\left(||z_{l,i}||_2 ||g_l||_2\right)} \quad \text{(S3)}$$

Because TRAST is intended to highlight local token distinctiveness, the implementation inverts the similarity:

$$q_{l,i} = -c_{l,i} \quad \text{(S4)}$$

Using -c_(l,i) is equivalent, after min-max normalization, to using 1 - c_(l,i). Therefore, larger values correspond to tokens whose feature directions differ more strongly from the global image descriptor.

### 1.5. Mathematical Rationale for Cosine Contrast

Cosine similarity isolates direction in embedding space and is therefore less sensitive to token-vector magnitude. This is useful for OCT heatmaps because scanner intensity, contrast rescaling, and local reflectivity can alter feature magnitudes without changing the diagnostic direction encoded by the representation. For L2-normalized token and global vectors, inverse cosine similarity is directly related to squared Euclidean distance:

$$|| z\_hat\_(l,i) - g\_hat\_l ||\_2\hat{}2 = 2(1 - c\_(l,i)) \quad \text{(S5)}$$

Thus, TRAST can be viewed as a normalized token-distance map in the learned representation space. A high value marks a token whose embedding is far from the dominant image-level descriptor for that block. In OCT, such locally distinctive tokens may correspond to fluid spaces, drusen, disruption of retinal layers, hyperreflective material, atrophic boundaries, or other morphology that differs from the surrounding scan structure.

The transformation from -c_(l,i) to 1 - c_(l,i) does not change the final map because min-max normalization is invariant to positive affine transformations:

$$mm(a\,u + b) = mm(u), for\ a > 0 \quad \text{(S6)}$$

### 1.6. Normalization and Spatial Reconstruction

Let q_l be the vector of all token contrast scores for block l. TRAST applies within-image min-max normalization. The implementation uses the following piecewise rule: if the block contains non-constant contrast values, scores are normalized to [0, 1]; otherwise, the block map is set to zero.

$$mm(q_{l,i}) = \frac{(q_{l,i} - q_{l,j})}{(q_{l,j} - q_{l,j})}, if\, q_{l,j} > q_{l,j}\ ;\ otherwise\ 0 \quad \text{(S7)}$$

The normalized vector is reshaped to the token grid inferred from N_l, then resized to the OCT input resolution using bilinear interpolation with align_corners = False:

$$M_l = U_{l\left(reshape(mm(q_l))\right)}, where\ M_l in\ [0,1]^{H\ x\ W} \quad \text{(S8)}$$

A second min-max normalization is applied after interpolation, again using the same constant-map safeguard. This preserves numerical comparability of maps across layers with different token-grid resolutions.

### 1.7. Early-Late Fusion

Swin Transformer features are hierarchical. Early blocks retain more local and edge-like retinal morphology, whereas late blocks encode more diagnosis-specific contextual patterns. TRAST therefore uses two sets of blocks:

E = {first two Swin blocks}, L = {last two Swin blocks} (S9)

For the SwinV2-base model used in the study, the flattened block list contains 24 transformer blocks arranged across four stages (2, 2, 18, and 2 blocks). Therefore E corresponds to the first two blocks and L corresponds to the final two blocks. The early and late maps are averaged separately:

$$M_E = \left(\frac{1}{|E|}\right) sum_{l\ in\ E} M_l, \quad M_L = \left(\frac{1}{|L|}\right) sum_{l\ in\ L} M_l \quad \text{(S10)}$$

The main TRAST heatmap fuses these maps as a convex combination:

$$H_{TRAST} = mm(alpha_E M_E + alpha_L M_L) \quad (S11)$$

The weights used in the study were:

alpha_E = 0.4, alpha_L = 0.6, alpha_E + alpha_L = 1 (S12)

This weighting preserves low-level anatomical localization while prioritizing the late-stage features most closely associated with the classifier's diagnostic representation.

### 1.8. TRAST Variants Used for Visualization

Two visualization variants were produced from the same mathematical scoring principle. TRAST version 1 displays the fused heatmap:

TRAST_v1(x) = H_TRAST(x) (S13)

TRAST version 2 displays the early and late maps separately, allowing readers to distinguish low-level local morphology from late-stage diagnostic abstraction:

TRAST_v2(x) = { M_E(x), M_L(x) } (S14)

The scoring rule, token extraction, inverse cosine contrast, and normalization steps are the same in both variants. The difference is only the display format.

### 1.9. Implementation Details Used in This Study

**Table S7.** TRAST implementation settings used for the OCT explainability experiments.

| Component | Setting used |
|---|---|
| Backbone | swinv2_base_window8_256.ms_in1k |
| Input size | 256 x 256 pixels |
| Hooked module | norm2 of each selected Swin block; fallback to norm1 or block output only if needed. |
| Selected blocks | First two and last two Swin blocks from the flattened block list. |
| Token map | Cosine similarity between each token and the block-level global descriptor. |
| Contrast direction | Similarity inverted before normalization, so lower similarity yields higher heatmap intensity. |

| | |
|---|---|
| Normalization | Per-block min-max normalization; constant maps set to zero. |
| Spatial resizing | Bilinear interpolation to the input OCT resolution with align_corners = False. |
| TRAST v1 fusion | 0.4 early map + 0.6 late map, followed by final min-max normalization. |
| TRAST v2 display | Early and late maps shown separately after independent normalization. |
| Probability reported | The predicted class and probability are obtained from the same forward pass used to capture tokens. |

### 1.10. Algorithmic Summary

**Table S8.** Algorithmic summary of TRAST for a single OCT B-scan.

| Step | Operation |
|---|---|
| 1 | Preprocess OCT image x and pass it through the trained Swin Transformer classifier. |
| 2 | Capture token descriptors from the selected early and late Swin blocks. |
| 3 | For each selected block l, compute global descriptor g_l as the mean of all token descriptors. |
| 4 | Compute token cosine similarities c_(l,i) between each token and g_l. |
| 5 | Invert similarity to obtain token contrast scores q_(l,i) = -c_(l,i). |
| 6 | Min-max normalize the token scores within each block. |
| 7 | Reshape the normalized token scores to the block token grid and upsample to the OCT input resolution. |
| 8 | Average early maps to obtain M_E and late maps to obtain M_L. |
| 9 | For TRAST v1, fuse maps as 0.4 M_E + 0.6 M_L and normalize. For TRAST v2, display M_E and M_L separately. |

### 1.11. Interpretation of the TRAST Heatmap

For a pixel location r in the final heatmap, higher TRAST intensity indicates that the corresponding spatial token, or the upsampled region derived from that token, is more dissimilar to the dominant token representation of the same image:

H_TRAST(r) high => token features near r are locally distinctive relative to the global OCT representation (S15)

This interpretation is especially appropriate for OCT B-scans because many diagnostically relevant findings are local departures from the expected retinal architecture: fluid compartments, drusen elevations, macular holes, layer discontinuities, or other focal changes. The method therefore emphasizes morphologic contrast in learned feature space rather than raw pixel brightness alone.

### 1.12. Relation to Model Decision and Faithfulness

TRAST is computed from the trained classifier's hidden representations during the same forward pass that produces the predicted class. It is therefore model-specific and image-specific. However, TRAST does not use class gradients and should not be described as a formal causal attribution method. Its role in this study is to provide an anatomically interpretable representation-space saliency map that can be judged by retinal specialists for clinical plausibility.

The use of normalized within-image token contrast also explains why the method is suitable for reader scoring: maps are displayed on a consistent [0, 1] scale, and the highest values identify the most locally distinctive model-internal regions for each OCT image.

**Algorithm S1. Token contRAST (TRAST) heatmap generation for one OCT B-scan**

**Input:**

Preprocessed OCT B-scan $x$ resized to $H \times W$ pixels; trained Swin Transformer classifier $f\theta$; selected early Swin blocks $E$; selected late Swin blocks $L$; fusion weights $\alpha$E = 0.4 and $\alpha$L = 0.6.

**Output:**

Final TRAST heatmap $H$TRAST resized to $H \times W$ pixels.

**Procedure:**

1. Register forward hooks on the selected Swin Transformer blocks. In the present implementation, the preferred hook is the *norm2* module, with *norm1* or the block output used only as fallback.

2. Perform a forward pass of $x$ through $f\theta$. Store the predicted class and the token descriptors captured from each selected block.

3. For each selected block $l \in E \cup L$:

3.1. Obtain the captured token descriptor matrix $Zl$. If the output is a feature grid, flatten it into a token matrix $Zl = \{zl,1, zl,2, \ldots, zl,Nl\}$ where $Nl$ is the number of spatial tokens.

3.2. Compute the block-level global descriptor $gl$ = mean($Zl$)

3.3. Compute the cosine similarity between each token $zl,i$ and the global descriptor $gl$.

3.4. Invert the similarity scores to obtain token contrast scores:

$ql,i = -$ cosine_similarity($zl,i$, $gl$)

3.5. Min–max normalize the token contrast scores within the block. If the map is constant, set the normalized map to zero.

3.6. Reshape the normalized scores to the spatial token grid of block $l$.

3.7. Upsample the block map to the OCT input size $H \times W$ using bilinear interpolation.

3.8. Apply a second min–max normalization after interpolation, again using the same constant-map safeguard.

3.9. Store the resulting block-level map as $Ml$.

4. Compute the mean early map:

$ME$ = average of $Ml$ for all $l \in E$

5. Compute the mean late map:

$ML$ = average of $Ml$ for all $l \in L$

6. Generate the fused TRAST heatmap:

$HTRAST$ = min–max normalize($0.4ME + 0.6ML$)

7. Return $HTRAST$.
